# Empagliflozin-Associated Euglycemic Diabetic Ketoacidosis in a Patient With Type 2 Diabetes Mellitus

**DOI:** 10.7759/cureus.33892

**Published:** 2023-01-17

**Authors:** Sukhjinder Chauhan, AndreyI Manov, Gundip S Dhillon, Pinak Shah

**Affiliations:** 1 Internal Medicine, Mountainview Hospital, Las Vegas, USA

**Keywords:** sodium-glucose cotransporter-2 (sglt-2) inhibitor, ketones, diabetes mellitus, empagliflozin, canagliflozin, sodium-glucose cotransporter-2 (sglt2) inhibitors, dka, euglycemic diabetic ketoacidosis

## Abstract

Euglycemic diabetic ketoacidosis (euDKA) is an uncommon condition, which is characterized by an elevated anion gap metabolic acidosis with ketonemia/ketonuria, in the presence of normal blood glucose levels. Common risk factors for the development of this condition include pregnancy, prolonged fasting, acute pancreatitis, and bariatric surgery. Sodium-glucose cotransporter-2 (SGLT-2) inhibitors have been identified as a rare cause of euDKA. A recent literature review on PubMed found only 86 case reports of euDKA secondary to SGLT inhibitors published in the medical literature up to December 2022. Here, we present the case of a 43-year-old man who was taking empagliflozin, an SGLT-2 inhibitor. The patient was found to have euDKA, which was likely an adverse effect of his medication.

## Introduction

Diabetic ketoacidosis (DKA) is a potentially life-threatening complication of diabetes mellitus (DM), which is caused by a reduction in the effective circulating insulin with a concurrent increase in counterregulatory hormones. DKA typically occurs in patients with Type 1 diabetes but can also develop in patients with Type 2 diabetes. It is characterized by a biochemical triad of hyperglycemia (blood glucose >250 mg/dL), elevated anion gap metabolic acidosis (arterial pH <7.3 and serum bicarbonate <15 mEq/L), and ketonemia (>3 mEq/L) or significant ketonuria.

However, a small subset of patients may develop elevated anion gap metabolic acidosis and ketonemia/ketonuria with blood glucose levels less than 250 mg/dL. This is known as euglycemic DKA (euDKA). The use of sodium-glucose cotransporter-2 (SGLT-2) inhibitors is an established but rarely reported risk factor for the development of this condition [1,2]. In this report, we describe the case of a patient with multivessel coronary artery disease (CAD) who was found to have euDKA secondary to SGLT-2 inhibitor therapy.

## Case presentation

A 43-year-old Asian man with a past medical history of myocardial infarction (MI), hypertension (HTN), and Type 2 diabetes mellitus (T2DM) initially presented to the Veterans Affairs (VA) hospital for intermittent chest pain which has been ongoing for several days. The patient stated that his chest pain was substernal, non-radiating, worsens upon exertion, and improves with rest. He reported that he had an MI in 2018 while he was living in Mexico. However, no further investigations and treatment, including percutaneous coronary intervention (PCI) were rendered at that time in 2018 because of financial reasons. He stated that he had moved back to the United States to seek better medical care. His home medications included atorvastatin, carvedilol, lisinopril, nitroglycerine, aspirin, and empagliflozin. At the VA hospital, the workup cardiac catheterization demonstrated multivessel Coronary artery disease (CAD). The patient was subsequently transferred to our hospital for possible coronary artery bypass grafting (CABG).

On arrival at our hospital, the patient was afebrile and tachycardiac with a heart rate (HR) of low 100s, and blood pressure (BP) was normal within the range of systolic BP of 120s and the diastolic BP of 70s. The patient was saturating 95% on 2 liters of oxygen via nasal cannula and was in no apparent respiratory distress. Complete blood count was unremarkable, while the chemistry panel shown in Table 1, was remarkable for a serum bicarbonate level of 18 mEq/L with an elevated albumin-adjusted anion gap of 15 mEq/L, the blood glucose level of 107 mg/dL, blood urea nitrogen (BUN) of 23 mg/dL, and serum creatinine level of 0.75 mg/dL. Hemoglobin A1c was 11.2%. Due to the elevated anion gap, serum lactic acid was obtained, which was unremarkable with a level of 1.3 mmol/L. Urinalysis shown in Table 2 was remarkable for a large number of ketones with elevated urine glucose of 1,000 mg/dL. Urinalysis was negative for bacteriuria or leukocyte esterase. These laboratory findings were consistent with a diagnosis of euglycemic diabetic ketoacidosis.

**Table 1:** Results of Chemistry Metabolic Panel (CMP) on admission.

Comprehensive metabolic panel	Results (reference)
Sodium	137 mmol/L (135–45)
Potassium	4.3 mmol/L (3.5–5.5)
Chloride	104 mmol/L (93–107)
Carbon Dioxide	18 mmol/L (21–32)
Anion GAP	15 mmol/L (4–12)
Blood Urea Nitrogen (BUN)	23 mg/dL (7-18)
Creatinine (Cr)	0.77 mg/dL (0.52-1.23)
Albumin	4.1 g/dL (6.4-8.3)
Lactic Acid	1.3 mmol/L (0.4-2.0)
Glucose	107 mg/dL (70-110)
Hemoglobin A1c (HbA1c)	11.2 % (4.2-6.3)

**Table 2:** Urinalysis results were positive for large ketones.

Urinalysis	Results (Normal reference)
Urine Color	Yellow (Yellow)
Urine Appearance	Clear (Clear)
Urine pH	5.5 (5.0-9.0)
Urine Protein	Negative mg/dL (Negative)
Urine Nitrites	Negative (Negative)
Urine Leukocyte Esterase	Negative (Negative)
Urine Glucose	1000 + mg/dL
Urine Ketones	LARGE

**Figure 1:**
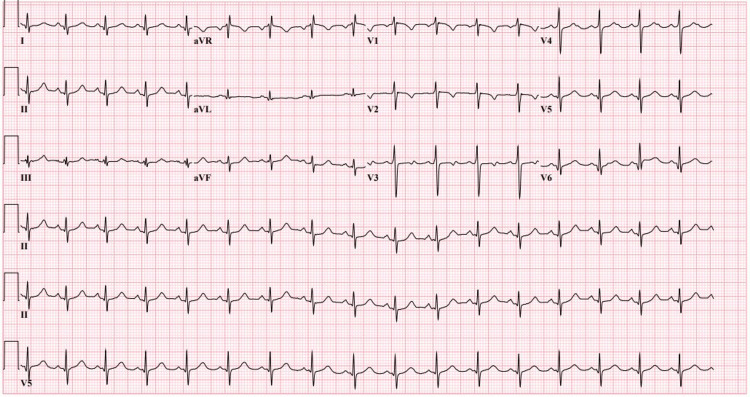
Electrocardiography (EKG) demonstrating Sinus Tachycardia, and T-Wave inversions in the anterior leads.

The patient’s high-sensitivity troponins were elevated (>600 ng/L). Electrocardiography (EKG) in Figure 1, demonstrated T-wave inversion in lateral leads and possible infarct on the anterior leads.

Echocardiography of the heart demonstrated an estimated left ventricular ejection fraction (LVEF) of 55 %. Doppler parameters were consistent with abnormal left ventricular relaxation (Grade 1 diastolic dysfunction). There was severe hypokinesis of the apical wall. Coronary angiography imaging results obtained from the VA hospital demonstrated multivessel disease with high-grade stenosis involving the left anterior descending artery (LAD), left circumflex artery (LCX), right coronary artery (RCA), and LV apical hypokinesis. Given the patient’s young age and Asian ethnicity, erythrocyte sedimentation rate (ESR), C-reactive protein (CRP), and complement levels (C3, C4) were ordered as shown in Table 3, which were unremarkable. Pathology samples for radial artery and right coronary artery plaque were negative for any specific pathologic changes which ruled out possible Kawasaki disease or any other form of vasculitis.

**Table 3:** Immunology panel results to rule out possible Kawasaki disease.

Immunology Panel	Results (reference)
Erythrocyte Sedimentation Rate (ESR)	18 MM/HR (0-15)
C-reactive Protein (CRP)	0.33 mg/dL (<0.90 mg/dL for inflammatory)
Complement C3 (C3)	153 mg/dL (90–180 mg/dL)

The patient was started on aspirin, heparin, statin, and lisinopril. Empagliflozin was held and a mild sliding scale insulin (SSI) via subcutaneous (SQ) route was initiated. After the empagliflozin was held and initiation of SSI, the patient's blood glucose was noted to be in the range of 107 mg/dL to 191 mg/dL during the hospitalization. 

**Table 4:** Daily blood glucose levels. This table shows the results of blood glucose levels on daily morning labs after the discontinuation of empagliflozin and initiation of sliding scale insulin (SSI) via the subcutaneous (SQ) route.

Blood Glucose levels	Results (reference: 70-110 mg/dL)
Day 1 ( day of Admission)	107
Day 2	118
Day 3	177
Day 4	119
Day 5	143
Day 6	87
Day 7	141
Day 8	182
Day 9	146
Day 10	191
Day 11	140
Day 12	160
Day 13	221
Day 14	141
Day 15 (Day of discharge)	146

The patient had a successful pump-support CABG with the left internal mammary artery (LIMA) to left anterior descending (LAD) artery, reverse saphenous vein graft to the diagonal artery, radial artery to obtuse marginal (OM) system, reverse saphenous vein graft to posterior descending coronary artery (PDA), with bypass to LAD, first obtuse marginal artery (OM1), second obtuse marginal artery (OM2), and right coronary artery (RCA). Following the procedure, the patient remained hemodynamically stable and had no post-operative (post-op) complications.

The endocrinology team was consulted for the further management of T2DM and for the adjustment of diabetic medications prior to discharge. Based on the relatively young age of the patient and the presence of euglycemic DKA, an immunology panel was ordered shown in Table 4, to exclude the possibility of Type 1 DM.

**Table 5:** Immunology panel results to exclude the possibility of T1DM. T1DM: Type 1 Diabetes Mellitus

Immunology Panel	Results (reference)
Islet cell Antibodies (Ab) Screen	Negative (Neg: <1:1)
Glutamic Acid Decarboxylase Antibody (GAD Ab)	<5.0 Units/mL (0.0–5.0 Units/mL)
C-Peptide	1.7 ng/mL (1.1–4.4 ng/mL)

The workup included C-peptide, islet cell antibodies, IA-2, ZnT-8, and glutamic acid decarboxylase-65 antibodies. The results were within normal limits, which confirmed the diagnosis of T2DM. The patient was discharged on aspirin 81 mg, clopidogrel bisulfate 75 mg, atorvastatin 80 mg, metoprolol tartrate 24 mg BID (twice a day), insulin glargine 8 Units at bedtime as a part of a bolus regimen, and correction dose rapid-acting insulin before meals, and oxycodone/acetaminophen 5/325 mg. The SGLT-2 inhibitor, empagliflozin, was discontinued indefinitely. The patient was recommended to follow up outpatient with the endocrinology clinic for further monitoring of T2DM and dose adjustment of medications as needed.

## Discussion

Euglycemic diabetic ketoacidosis (euDKA) is an infrequent complication of diabetes, which is characterized by severe ketoacidosis in the presence of normal or near-normal blood glucose levels (<250 mg/dL). Some of the risk factors for the development of euDKA include pregnancy, prolonged fasting, surgery, acute pancreatitis in patients without DM, glycogen storage disorders, chronic liver disease, gastroparesis, and insulin pump failure. Sodium-glucose cotransporter-2 (SGLT-2) inhibitors have also been identified as an uncommon risk factor for the development of this condition [3-6].

SGLT-2 inhibitors are a class of oral antihyperglycemic agents, which exert their effect on the SGLT-2 proteins expressed in the proximal convoluted tubules of the kidneys. These proteins normally reabsorb the filtered glucose from the tubular lumen. By inhibiting these proteins, the SGLT-2 inhibitors prevent the reabsorption of filtered glucose, thus, lowering the renal threshold for glucose and increasing urinary glucose excretion. At present, four SGLT-2 inhibitors have been approved by the United States Food and Drug Administration (USFDA). These include canagliflozin, dapagliflozin, empagliflozin, and ertugliflozin. The clinical indications for each of these medications vary; however, all four of them have been approved for the management of T2DM in adult patients. They are typically used as second or third-line antihyperglycemic agents but can also be used as monotherapy if a contraindication to the use of metformin is present. They are also indicated in patients with T2DM and atherosclerotic cardiovascular disease, patients with or without T2DM and congestive heart failure (CHF) with preserved or reduced ejection fraction, and patients with T2DM and renal disease such as diabetic nephropathy [5,7].

SGLT-2 inhibitors have also been associated with additional beneficial effects. For instance, in patients with multivessel CAD and diabetes, as was the case with our patient, CABG has been found to be superior to PCI, as it leads to better survival rates and a lower risk of myocardial infarction and repeats re-vascularizations [8,9]. In diabetic patients treated with CABG, SGLT-2 inhibitors have been found to cause a significant reduction in cardiovascular and all-cause mortality, and hospitalization rates for heart failure. For instance, in the EMPA-REG OUTCOME trial, 7,028 patients with a history of T2DM and established cardiovascular disease (e.g., multivessel coronary artery disease, single-vessel coronary artery disease, peripheral artery disease, or a history of myocardial infarction or stroke) were randomized. Among these patients, 1,738 patients had a history of CABG. It was found that in patients with T2DM and a history of CABG at baseline, empagliflozin reduced the risk of cardiovascular mortality by 48%, hospitalization for heart failure by 50%, and death from any cause by 43% [10]. Similarly, these agents have also been associated with a reduction in both systolic and diastolic blood pressure from baseline. Results from the EMPA-REG BP trial showed a reduction of 3.44 mmHg in systolic blood pressure and a 1.36 mmHg reduction in diastolic blood pressure from baseline in patients who received empagliflozin 10 mg. Patients who received 25 -mg of empagliflozin showed a reduction of 4.16 mmHg and 1.72 mmHg in the systolic and diastolic blood pressure from their baseline, respectively [11]. Studies have also shown SGLT-2 inhibitors to be renal protective agents, which can cause a reduction in the progression of albuminuria in patients with diabetic nephropathy, doubling of the serum creatinine, development of end-stage renal disease (ESRD), and renal death. The composite renal outcome was reduced by 46% in patients receiving empagliflozin in the EMPA-REG OUTCOME study [12].

These beneficial effects make SGLT-2 inhibitors an attractive class of oral antihyperglycemics for the treatment of Type 2 diabetes. However, it is important to note that these medications are associated with an initial decline in the glomerular filtration rate (GFR), and thus, should not be initiated in patients with a very low GFR. An eGFR <30 mL/min/1.73m^2^ is considered to be an absolute contraindication for all four agents [7]. However, more recent studies have shown that SGLT-2 inhibitors may be safe to use in patients with heart failure down to an eGFR of 20 ml/min/1.73m^2^ [13]. 

SGLT-2 inhibitors are associated with several adverse effects, such as genital mycotic infections, urinary tract infections, increased urination, back pain, and dyslipidemia [5]. Euglycemic DKA is a rare yet serious side effect of these medications. A recent literature review found only 77 case reports of euDKA secondary to SGLT inhibitors published in the medical literature up to 02 August 2020. The findings of this review showed that almost half of the patients who developed euDKA had Type 2 diabetes without any comorbidities. T2DM with concurrent cardiovascular disease was noted to be present in about one in five patients, while one in 10 patients was a Type 1 diabetic (T1DM). The most common presenting complaints included nausea, vomiting, abdominal pain, and shortness of breath; however, the symptoms were found to be lesser as compared to classical DKA. Surgery was the most prevalent precipitating factor for euDKA induced by SGLT-2 inhibitors. Infections and cardiovascular irregularities were the other precipitating factors [14]. Our search on PubMed revealed 86 case reports of euDKA associated with SGLT-2 inhibitors until December 2022.

The pathophysiology of euDKA secondary to SGLT-2 inhibitors is thought to be caused by a decrease in insulin production and an increase in glucagon secretion, which leads to gluconeogenesis, glycogenolysis, lipolysis, and ketogenesis [14]. In normal conditions, the influx of glucose into the pancreatic alpha cells increases the ratio of ATP (adenosine triphosphate) to ADP (adenosine diphosphate). This leads to a decreased efflux of potassium and a decreased influx of calcium, along with a decrease in the secretion of glucagon from pancreatic alpha cells. Under the effect of SGLT-2 inhibitors, the glucose influx into pancreatic alpha cells is also reduced. This leads to a decrease in the ATP/ADP ratio, an increase in the efflux of potassium, and an increase in the influx of calcium from pancreatic alpha cells. The increased influx of calcium leads to increased secretion of glucagon, the major ketogenic hormone, from the alpha cells, resulting in an increased glucagon/insulin ratio, which favors lipolysis and ketogenesis. This also results in a shift of glucose to fat metabolism and causes the production of ketones [15]. Simultaneously, SGLT-2 inhibitors increase the urinary excretion of glucose, thus, lowering blood glucose levels. This in turn causes a decrease in the secretion of insulin from the pancreatic beta cells, which results in excess ketone body formation. Moreover, the inhibition of SGLT-2 transporters generates a positive electrochemical gradient, which causes an increase in the renal reabsorption of ketone bodies, thus increasing serum ketone levels [14,16]. 

The development of SGLT-2 inhibitor-induced euDKA may be prevented by withholding these medications during any situation that may be associated with an increased risk of precipitating this condition, such as acute illness, surgery, acute pancreatitis, and dehydration. Based on the long half-life of these agents, it is recommended that they should be stopped at least three days prior to a major surgical procedure [16,17]. In patients who develop euDKA due to an SGLT-2 inhibitor, management involves the immediate cessation of the drug and initiation of the DKA protocol, which includes the administration of intravenous fluids and insulin [16,18]. Our patient presented to us for the evaluation of CAD and was asymptomatic but was incidentally found to have euDKA. He had normal C-peptide levels and was negative for islet cell antibodies, IA-2 antibodies, XnT-8 antibodies, and glutamic acid decarboxylase-65 antibodies, which ruled out Type 1 diabetes. His normal blood glucose levels, massive ketonuria, and a normal lactic acid level with a history of empagliflozin use led us to the diagnosis of SGLT-2 inhibitor-induced. We believe that our patient’s history of coronary artery disease and myocardial ischemia may have resulted in an increase in stress hormones and promoted the development of euDKA, which is consistent with previously reported cases [19,20].

## Conclusions

SGLT-2 inhibitors, such as empagliflozin, are novel antihyperglycemics that lower blood glucose levels by blocking renal tubular reabsorption of glucose. Euglycemic DKA (euDKA) is a rarely reported yet serious side effect associated with these medications. It presents with severe ketoacidosis but with normal blood glucose levels, and thus, may easily be missed, resulting in diagnostic and treatment delays. A delay in the treatment of euDKA can lead to severe complications, such as dehydration, hypovolemic shock, seizures, coma, and death. Therefore, it is of great importance that physicians are aware of this uncommon condition, so that it can be recognized and treated in a timely manner.
